# TME14/457: Teleradiology: Opinion and technical requirements of German radiologists

**DOI:** 10.2196/jmir.1.suppl1.e121

**Published:** 1999-09-19

**Authors:** M Walz, C Brill, R Bolte, K J. Lehmann, T Hothorn, M Georgi

**Affiliations:** ^1^Institut für klinische Radiologie, Klinikum Mannheim, MannheimGermany

**Keywords:** Teleradiology, Telemedicine

## Abstract

**Introduction:**

For the evolution and acceptance of solutions in telemedicine - concerning e. g. liability, economics, security, medical and technical quality - it is very important to learn what the opinion and concepts of the present and future users - the medical professionals - are.

**Methods:**

In 1997 a questionnaire was sent to 4400 German radiologists in hospitals and private offices with a response rate of 5 %. Intensive statistical analysis has been performed by SAS. The survey has been funded by the German government represented by BMBF and DFN. To evaluate the changes in the opinion and technical requirements a second questionnaire was sent to 1500 radiologists in May 1999.

**Results:**

The results showed that in 1997 only 47 % of responders felt well informed about teleradiology. 83% of the radiologists use PC, 52% have installed workstations, 33% use the DICOM 3.0 Standard, PACS are installed in 14 % of the institutions. In the opinion of German radiologists its main future application areas will be the emergency and expert consultation, but - more and more - radiologist services were expected to be provided from home or central offices too. Image and report transfer plus common telemedicine integration as well as interfaces to reference databases, educational applications, technical quality surveillance and product support (maintenance) were considered to be increasingly important areas. Smaller institutions judged expert consultation as more important than bigger institutions. Technical standardization and system stability were strongly demanded. From the medico-legal point of view, there was a demand for as strong as possible an association between radiological report and image, appropriate data security, and solutions of liability questions as well as for guidelines, e. g. of correct documentation and necessary image quality. Links to RIS and PACS were considered especially important for those who already work with these systems. The introduction of fair payments was mainly a matter of the radiologists in private consulting rooms. Most of radiologists thought that lossy compression should be allowed if no loss of relevant information occurs.

**Discussion:**

Technically most demands can be fulfilled today but are not yet commonly included in teleradiology or telemedicine systems. Other aspects as legal or financial requirements must be discussed and solutions provided before the majority will use telemedicine. It must be recognized that there exist many different application areas with different requirements. Local conditions and interests of radiologists - and other medical or non-medical groups - are different too.

